# Comparison between kinetic modelling and graphical analysis for the quantification of [^18^F]fluoromethylcholine uptake in mice

**DOI:** 10.1186/2191-219X-3-66

**Published:** 2013-09-14

**Authors:** Dominique Slaets, Filip De Vos

**Affiliations:** 1 Laboratory of Radiopharmacy, Faculty of Pharmaceutical Sciences, Gent University, Harelbekestraat 72, 9000, Ghent, Belgium

**Keywords:** PET, [^18^F]Fluoromethylcholine, Kinetic modelling, Patlak plot, Graphical analysis, Metabolic trapping

## Abstract

**Background:**

Until now, no kinetic model was described for the oncologic tracer [^18^F]fluoromethylcholine ([^18^F]FCho), so it was aimed to validate a proper model, which is easy to implement and allows tracer quantification in tissues.

**Methods:**

Based on the metabolic profile, two types of compartmental models were evaluated. One is a 3C2i model, which contains three tissue compartments and two input functions and corrects for possible [^18^F]fluorobetaine ([^18^F]FBet) uptake by the tissues. On the other hand, a two-tissue-compartment model (2C1i) was evaluated. Moreover, a comparison, based on intra-observer variability, was made between kinetic modelling and graphical analysis.

**Results:**

Determination of the [^18^F]FCho-to-[^18^F]FBet uptake ratios in tissues and evaluation of the fitting of both kinetic models indicated that corrections for [^18^F]FBet uptake are not mandatory. In addition, [^18^F]FCho uptake is well described by the 2C1i model and by graphical analysis by means of the Patlak plot.

**Conclusions:**

The Patlak plot is a reliable, precise, and robust method to quantify [^18^F]FCho uptake independent of scan time or plasma clearance. In addition, it is easily implemented, even under non-equilibrium conditions and without creating additional errors.

## Background

[^18^F]Fluoromethylcholine ([^18^F]FCho) and [^11^C]choline ([^11^C]Cho) are radiolabelled biomarkers to study the altered phospholipid metabolism in tumours. Several ongoing clinical trials evaluate their use as diagnostic [1] or as therapy response assessment [2,3] tools. The latter requires quantification of the tracer uptake. Thus far, this is executed in a semi-quantitative way using standardized uptake values (SUV) [4], the percent of injected dose per gram of tissue (%ID/g) [3], or by comparing tumour-to-muscle ratios [5]. SUV have an advantage in that they are straightforward to estimate, but when applied in a clinical setting, care should be given about standardization [6]. On the other hand, absolute quantification of tracer uptake, by means of kinetic modelling, is more complex since it requires dynamic scanning and arterial sampling. Nevertheless, this technique is more reliable and independent of scan time or plasma clearance, allowing a more accurate quantification of the tracer uptake [7].

**Figure 1:**
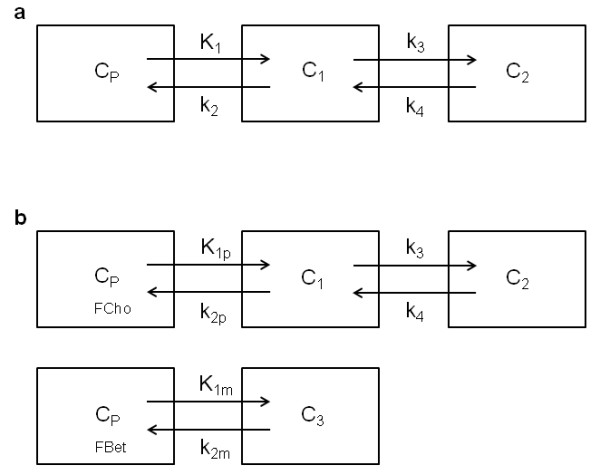
**Visual representation of the different kinetic models. (a)** Schematic of a two-tissue-compartment model with one input function (2C1i). Transfer coefficients *K*_1_ and *k*_2_ describe the uptake and washout of the free tracer and its metabolite ([^18^F]FBet) across cell membranes. Rate constant *k*_3_ is regarded as the conversion of [^18^F]FCho into [^18^F]FPCho, which is assumed to be metabolically trapped in the cell. Micro-parameter *k*_4_ represents dephosphorylation and is expected to be very small. **(b)** Schematic of a three-tissue-compartment model with two input functions (3C2i). Rate constants *K*_1p_ and *K*_1m_, or *k*_2p_ and *k*_2m_ represent respectively the uptake and washout of [^18^F]FCho and [^18^F]FBet.

Until now, no kinetic model was described for [^18^F]FCho, so it was aimed to validate a proper model, which is easy to implement and allows quantification of [^18^F]FCho uptake in tissues. The validation studies were performed on laboratory animals, i.e. mice, since no (fundamental) differences in choline metabolism between mice and human tissues were reported. Moreover, both species are characterized by a fast and explicit oxidative metabolism of [^18^F]FCho into the osmolyte [^18^F]fluorobetaine ([^18^F]FBet). Therefore, a test was performed to evaluate if this metabolite is characterized by a specific uptake in the tissues, eventually provoking overestimation of the [^18^F]FCho uptake in tissues. To verify this possibility, two types of compartmental models were evaluated (Figure 1): a 3C2i model which contains three tissue compartments and two input functions (one for [^18^F]FCho and one for [^18^F]FBet) and corrects for possible [^18^F]FBet uptake by the tissues, and a 2C1i model which contains two tissue compartments and one input function and quantifies the [^18^F]FCho uptake, based exclusively on [^18^F]FCho plasma concentrations. As kinetic modelling, using non-linear least squares optimisation, is computationally expensive and sensitive to noise, a comparison will be made between kinetic modelling and graphical analysis to quantify [^18^F]FCho uptake in tissues. The latter uses a linearization of the compartmental equations, resulting in a faster method which is less sensitive to noise [7].

## Methods

Athymic Swiss Nu/Nu mice (female, 20 g) were obtained from Charles River Laboratories International (Janvier, France). Sulphuric acid, urea, dimethylaminoethanol, chloroform, and methanol were purchased from Sigma-Aldrich (Steinheim, Germany). Physiological saline was obtained from B.Braun Medical Ltd. (Sheffield, UK). Isoflurane and heparin were obtained from Medini N.V. (Oostkamp, Belgium).

### Radiochemistry

[^18^F]FCho was prepared by nucleophilic substitution of dimethylaminoethanol to [^18^F]fluoromethylbromide as described by Slaets et al. [8].

### Positron emission tomography

All animal experiments were approved by the local ethics committee (ECD 11/30). Mice (*n* = 6) were harvested 8 h prior to the study and anaesthetized with isoflurane (1.5% + 1.5 L/min O_2_) during cannulation, animal handling, and acquisition time. Prior to positioning on the heated scanner bed, the mice were venously and arterially cannulated for tracer injection and arterial blood sampling, respectively. Positron emission tomography (PET) was performed using a Flex Triumph II μPet scan (Gamma Medica, Sherbrooke, Quebec, Canada) with a 7.5-cm axial field of view and a 1.2-mm spatial resolution. After the synchronized start of the arterial blood sampling and the start of the μPET acquisition, the tracer was intravenously injected (±11.1 MBq in 150 μL saline) over a constant infusion interval of 5 s. List mode data were acquired for 40 min.

### Arterial input function determination

#### Blood concentration

Continuous arterial blood samples were withdrawn for 40 min at a rate of 10 μL/min using a microvolumetric blood counter (μBC, Gamma Medica-Ideas) as described by Convert et al. [9]. Heretofore, a 60-cm PE10 catheter filled with heparinized saline (50 U/mL) was inserted in the carotid artery and the withdrawing syringe was placed at the end. At a distance of 20 cm from the mouse, the catheter was placed in the calibrated detector unit of the μBC. The obtained blood curve (*g*(*t*)) was automatically corrected for decay and propagation delay. Corrections for dispersion were performed using Equation 1:

(1) $$C_{\mathrm{bl}}\left(t\right)=g\left(t\right)+\tau_{\text{disp}}\times \frac{\text{dg}}{\text{dt}}.$$

In Equation 1, *g*(*t*) depicts the original blood curve, $\frac{\text{dg}}{\text{dt}}$ describes the first derivative of the original blood curve, *τ*_disp_ is a constant calculated as described by [9], and *C*_bl_ represents the for dispersion-corrected blood curve.

#### Plasma concentration and metabolites

The ratio of the tracer plasma to blood concentration and metabolite profile was determined *ex vivo* in Swiss NuNu mice (*n* = 4 per time point) by an intravenous injection of 22.2 MBq [^18^F]FCho in 150 μL saline. Blood (200 μL) obtained by cardiac puncture in the left heart ventricle at 0.5, 2.5, 10, 20, and 40 min post injection was collected in heparinized tubes and spiked with 5% sulphuric acid (100 μL) and a 40% urea solution (100 μL) before centrifugation (5 min, 3,000×*g*). Plasma and the hematocrit pellet were separated and assessed for radioactivity. Then, 600 μL of the Bligh and Dyer extraction solution containing CHCl_3_:MeOH:H_2_O (1:2:1) was added to the supernatant (200 μL) of all time points except that of 0.5 min (post injection) p.i. and mixed vigorously before centrifugation (5 min, 3,000×*g*). Finally, the two layers were separated and assessed for radioactivity, and 500 μL of the aqueous layer was injected to a semi-preparative high-performance liquid chromatography (HPLC) system for aqueous metabolite analysis as described by Bansal et al. [10]. The HPLC system consisted of a Waters 1525 binary pump (Waters, Milford, MA, USA), a Waters Breeze data acquisition, and an Alltima silica NP column (5 μm, 10 × 250 mm). The following gradient elution [10] was used at a rate of 7 mL/min. The retention time of [^18^F]FCho was determined by extraction (*vide supra*) of the reference solution and was 14.4 min. The metabolite [^18^F]FBet had a retention time of 12.4 min in the chromatographic separation. During separation, fractions of 20 s were collected and assessed for radioactivity. The tracer plasma-to-blood concentration ratio was calculated as (Equation 2)

(2) CplCbl=CPSpl%pli.e.0.60CPSbl%bli.e.1.00

In Equation 2, the ratio of the plasma to blood concentration is calculated for several time points (*vide supra*). The plasma concentration (*C*_pl_) was determined by dividing the counts in the plasma (CPS_pl_) to the standardized volume of plasma in blood (%pl, which is expressed as 0.6). In analogy, the tracer blood concentration (*C*_bl_) was calculated by taking the ratio of the sum of the counts in the plasma and hematocrit pellet (represented by CPS_bl_) to the blood volume, which is expressed as 1.

The obtained ratios and metabolite percentages were fitted to a single-phase exponential curve. The goodness of the fit and the equation were calculated using GraphPad Prism 5.00 for Windows (San Diego, CA, USA; http://www.graphpad.com).

### Image reconstruction and corrections

Dynamic images were reconstructed using the MLEM 2D algorithm with ten iterations to the following time frames: 12 s × 5 s, 6 s × 10 s, 6 s × 20 s, 6 s × 60 s, 4 s × 120 s, 3 s × 300 s, and 1 s × 360 s. Quantification of the PET scan was performed relative to a ^22^Na source placed on the base of the scanning bed.

### Regional time-activity curve computation

The last time frame was used to draw a region of interest on two to three adjacent slices on a part of the tissue of interest, namely the brain, kidney, lung, liver, and muscle. The ROIs were then placed over the complete series of time frames to derive tissue time-activity curves.

### Kinetic modelling

The exploit of kinetic modelling to [^18^F]FCho is based on the following general assumptions [11,12]:

There is a single source for [^18^F]FCho, i.e. the plasma. The concentration varies with time, and transfer from the plasma to the first tissue compartment is reversible.

The capillary concentration of [^18^F]FCho is assumed to be equal to the arterial plasma concentration, so no gradient could be observed across the capillary membranes.

Since tracer amounts were injected (high specific activity), the test solute did not alter the system and first-order kinetics could be assumed.

[^18^F]FCho is homogeneously distributed in the tissues.

Metabolism of choline is under steady state, so the concentration of phosphocholine stays constant during imaging.

In addition to the previous assumptions, [^18^F]FCho is metabolized to (1) [^18^F]fluorophosphocholine which is suspected to be metabolically trapped and (2) [^18^F]FBet, an osmolyte, which is able to cross the cell membranes.

Kinetic analysis was performed using a 3C2i as described by Fujita et al. [13] and was weighed against a 2C1i (Figure 1). The models are described by following the micro-parameters: *K*_1_, *k*_2_, *k*_3_, and *k*_4_ for the 2C1i model; and the additional parameter *K*_1p_/*K*_1m_ which represents the ratio of [^18^F]FCho to [^18^F]FBet uptake in tissues in the 3C2i model. Transfer coefficients *K*_1_ and *k*_2_ describe the uptake and washout of the free tracer and its metabolite ([^18^F]FBet) across cell membranes. Rate constant *k*_3_ is regarded as the conversion of [^18^F]FCho into [^18^F]FPCho, which is assumed to be metabolically trapped in the cell. Micro-parameter *k*_4_ represents dephosphorylation and is expected to be very small. The 3C2i model differentiates between [^18^F]FCho and [^18^F]FBet uptake or washout by rate constants *K*_1p_ and *K*_1m_, or *k*_2p_ and *k*_2m_, respectively. During the fitting process using the 3C2i model, six parameters were identified. The vascular volume parameter, present in both models, was not accounted for due to the fast clearance of [^18^F]FCho. Parameters of interest were the ratio of [^18^F]FCho to [^18^F]FBet uptake in tissues, represented by *K*_1p_/*K*_1m_ for the 3C2i model, and the [^18^F]FCho influx across the membranes (*K*_i_) calculated as *K*_1_*k_3_/(*k*_2_ + *k*_3_) [14-16] for both models.

Before the selected kinetic models were fitted, the tracer plasma curves were fitted using a three-exponential decay function [17]. Then, model fitting was conducted and optimized by (1) visual inspection of the agreement of time-activity curve (TAC) and the model output, (2) evaluation of the percent coefficient of variation of the micro-parameters, and (3) the goodness of fit displayed by the Akaike information criterion (AIC), Schwartz criterion (SC), and model selection criterion (MSC) as determined by the PMOD software (PMOD version 3.0; PMOD group, Zurich, Switzerland). When the fitting was poor, fits were redone by adjusting the initial parameters. Parameter estimation, in kinetic modelling, was performed using the Marquardt-Levenberg algorithm, which uses a non-linear least squares optimization process, included in the PMOD software.

### Graphical analysis

Since absolute quantification of [^11^C]Cho was already established by means of the Patlak plot [12,17], this graphical analysis was also applied for [^18^F]FCho. The macro-parameter of interest, i.e. the slope, which resembles [^18^F]FCho influx into the cells, was determined under non-equilibrium and equilibrium conditions. The latter is usually achieved when the graphical plot describing normalized tissue concentration as a function of the normalized time becomes linear [11,12]. It is worth noting that the Patlak slope corresponds to the macro-parameter of interest *K*_i_, defined previously during the kinetic modelling process.

### Intra-observer variability of macro-parameters

A test was performed to evaluate the robustness of the macro-parameter estimation of the [^18^F]FCho influx (i.e. *K*_i_) (*vide supra*) using these three models, i.e. 3C2i, 2C1i, and the Patlak plot. The percent error is calculated as (Cho influx test − Cho influx retest/(mean test and retest).

### Statistical analysis

Statistical analysis was performed using Graphpad Prism 5.00. A D'Agostino-Pearson test was conducted to test for normality distribution of the data. Since samples were too little, non-parametric statistical tests were used. A Bland-Altman plot was determined to evaluate the percent error during the intra-observer variability test. A Kruskal-Wallis test was performed to evaluate statistical differences between (1) the AIC, SC, and MSC fitting factors of the different models evaluated and (2) the determined [^18^F]FCho influxes across the cell membrane by comparing the macro-parameter *K*_i_. A Wilcoxon signed-rank test was applied to evaluate if the median ratio *K*_1p_/*K*_1m_, which represents the ratio of [^18^F]FCho to [^18^F]FBet uptake in tissues in the 3C2i model, was statistically different from 1. A Spearman correlation test was performed to evaluate the influence of equilibrium conditions on the model's Patlak slope. Differences were regarded as statistically significant for *p* < 0.05.

## Results

### Arterial input function determination

Since kinetic modelling requires an arterial plasma input function of the unmetabolized tracer, corrections are forced on the blood curve which was obtained by the microvolumetric blood counter. The corrections applied were dispersion correction, conversion of the blood curve into a plasma curve, and corrections for metabolites.

#### Dispersion

**Figure 2:**
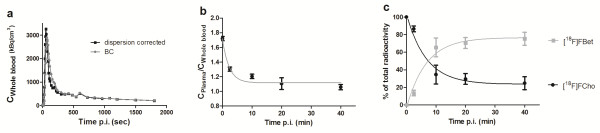
**Determination of the arterial input function. (a)** A time-activity curve determined by the blood counter (grey circles) and for the dispersion-corrected whole blood curve (black squares). **(b)** The single-phase exponential decay fitting of the ratio of the tracer plasma to blood concentration. Data points are expressed as mean ± S.D. **(c)** The single-phase exponential fitting of the percentages of [^18^F]FCho and [^18^F]FBet as a function of time after injection. Data points are expressed as mean ± S.D.

Based on the specification of tubing diameter (PE 10), withdrawal speed (10 μL/min), and distance to the detector unit (i.e. 20 cm), the *τ*_disp_ constant was calculated as 12.2092 s. All blood curves were first smoothed by calculating the average concentrations (kBq/cm^3^) in agreement with the μPET mid-time frames and then corrected for dispersion as described by Equation 1. An original blood curve and its corresponding dispersion-corrected blood curve are displayed in Figure 2a.

#### Plasma concentration

The ratio of the plasma to blood concentration was fitted by a single-phase exponential decay (Figure 2b) with *R*^2^ = 0.9374 and a corresponding equation (Equation 3):

(3) CplCbl=0.6007∗e−0.4559*timemin+1.116.

The plasma concentration was achieved by multiplying the obtained ratios for each time point by the dispersion-corrected blood curve as determined previously.

#### Corrections for metabolites and parent compound

Use of the Bligh and Dyer extraction mixture allowed estimation of the percentage of lipophilic metabolites, while the HPLC analysis of the hydrophilic fraction allowed identification and quantification of the amount of hydrophilic metabolites and parent compound in the plasma. The fraction of lipophilic compounds in the plasma was lower than 5% for all time points (Table 1); therefore, no further corrections were made to correct for the share of these metabolites. On the other hand, only one hydrophilic metabolite, i.e. [^18^F]FBet, which appeared rather quickly, was identified in the plasma (Figure 2c). Indeed, after 10 min [^18^F]FBet already amounts to 58.54%. The measured percentages of [^18^F]FCho and [^18^F]FBet were fitted to a single-phase exponential curve respectively with Equations 4 and 5 and *R*^2^ = 0.9435.

(4) $$\%\;\left[{}^{18}F\right]\;\text{FCho}=0.8043\;*\;e^{-0.1497\;*\;\mathrm{time}\;\left(\min\right)}+\;0.2346$$

(5) $$\%\;\left[{}^{18}F\right]\;\mathrm{FBet}=0.8043\;*\;e^{-0.1498\;*\;\mathrm{time}\;\left(\min\right)}+\;0.7653$$

**Table 1:** **Percentage of lipophilic and hydrophilic [**^18^F**]FCho metabolites present in arterial plasma**

**Time p.i. (min)**	**Hydrophilic metabolites (%)**	**Lipophilic metabolites (%)**
2.5	99.60 ± 0.13	0.40 ± 0.13
10	98.64 ± 0.05	1.36 ± 0.50
20	99.31 ± 0.07	0.69 ± 0.07
40	97.09 ± 1.21	2.91 ± 1.21

The corresponding percentages per time point obtained from Equations 4 and 5 were multiplied by the plasma curves (*vide supra*) to obtain the input functions for the selected compartmental models, i.e. the 2C1i and 3C2i models. The [^18^F]FCho plasma concentration was the only input function in the 2C1i model, whereas the [^18^F]FCho plasma concentration and the [^18^F]FBet plasma concentrations were both used in the 3C2i model.

### Kinetic modelling

Prior to the mathematical modelling, the obtained input function was fitted to a three-exponential function. When the 3C2i and 2C1i models were fitted to the TACs, the goodness of fit was described by AIC, SC, and MSC parameters presented in Table 2. Comparing these parameters, no statistical differences were observed between the 3C2i and the 2C1i model (*p* values for the Kruskal-Wallis test were 0.9925, 0.8276, and 0.9342, respectively), albeit reducing the number of compartments accordingly decreased the percent coefficient of variance (%COV) on the micro-parameter estimates (data not shown).

**Table 2:** Fitting factors per tissue (AIC, SC, and MSC) for 3C2i and 2C1i models

	**AIC**		**SC**		**MSC**	
	**3C2i**	**2C1i**	**3C2i**	**2C1i**	**3C2i**	**2C1i**
Brain	90.54 ± 31.56	91.44 ± 21.48	98.72 ± 31.63	97.00 ± 21.68	1.36 ± 0.52	1.341 ± 0.425
Kidney	24.63 ± 34.18	19.40 ± 30.54	32.81 ± 34.23	25.19 ± 30.58	3.188 ± 0.634	3.292 ± 0.554
Liver	51.97 ± 50.19	61.03 ± 41.54	60.13 ± 50.16	66.68 ± 41.43	2.708 ± 1.308	2.544 ± 1.099
Lung	44.92 ± 24.72	47.79 ± 19.50	53.11 ± 24.75	49.06 ± 27.39	2.669 ± 0.616	2.600 ± 0.490
Muscle	92.96 ± 15.28	90.34 ± 15.73	100.5 ± 15.85	95.90 ± 15.93	1.489 ± 0.431	1.549 ± 0.333

**Figure 3:**
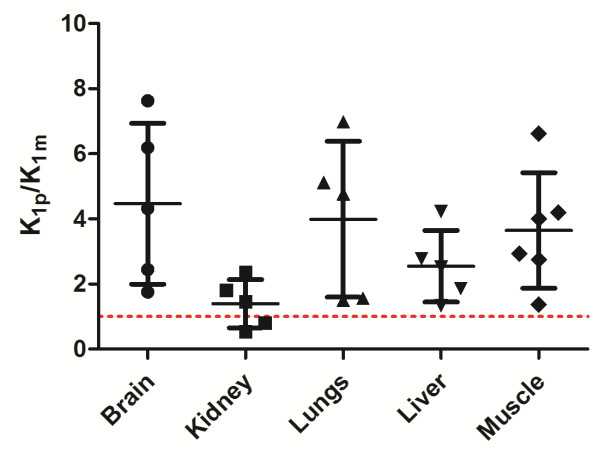
**Visual representation of the [**^**18**^**F]FCho-to-[**^**18**^**F]FBet uptake ratio in several tissues.** The [^18^F]FCho-to-[^18^F]FBet uptake ratio represented by *K*_1p_/*K*_1m_ was determined by the 3C2i model. The individual data points are shown, and the whiskers represent mean and S.D. The dotted line represents a ratio equal to 1.

**Figure 4:**
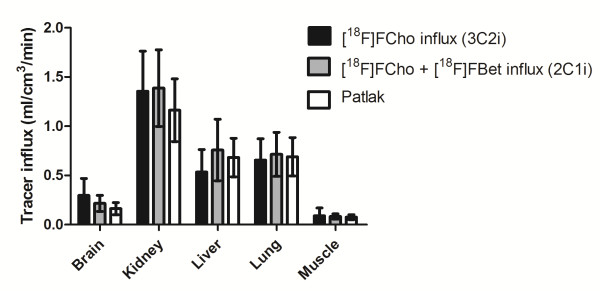
**Representation of the [**^**18**^**F]FCho influx determined by the different kinetic models.** The tracer influx (*K*_i_) was calculated as *K*_1_*k_3_/(*k*_2_ + *k*_3_) for the different tissues.

For each tissue, the mean ratio of [^18^F]FCho to [^18^F]FBet uptake in tissues (*K*_1p_/*K*_1m_) was larger than 1 (Figure 3). However, only the ratio of the muscle tissue was statistically different from 1 (Wilcoxon signed-rank test, *p* = 0.0313). Kidney tissue displayed the lowest ratio, indicating an elevated (or slightly higher) [^18^F]FBet uptake compared to the other tissues. It is worth noting that ratios with a %COV higher than 250% were treated as unreliable and were excluded from the dataset. When comparing the estimates of the [^18^F]FCho influxes across the cell membrane by *K*_i_ determination with or without corrections for possible [^18^F]FBet uptake (3C2i versus 2C1i model), no significant differences were observed for both models (Kruskal-Wallis test, *p* value = 0.8670) (Figure 4).

### Graphical analysis

Equilibrium conditions of the tracer were determined and were usually achieved between 1 and 23 min after injection. Then, linear regression was applied to the data with a good fit (Chi square, 0.0054505 ± 0.0049021) allowing estimation of the slope, which resembles the [^18^F]FCho influx into the cell. When comparing the Cho influxes *K*_i_ calculated for the 2C1i and 3C2i model with the slope of the Patlak plot, a good agreement was achieved and no statistical differences were observed (Kruskal-Wallis test, *p* = 0.8270) (Figure 4).

### Intra-observer variability of macro-parameters

**Figure 5:**
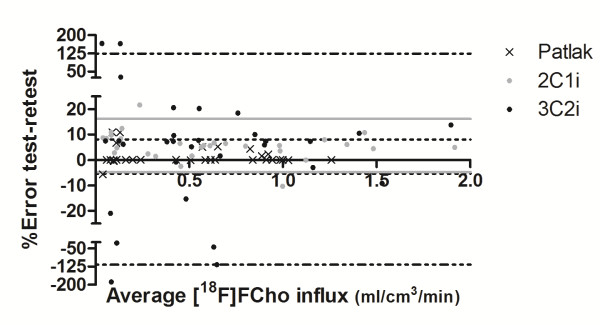
**A Bland-Altman plot representing the intra-observer variability of the macro-parameters *K***_**i **_**and Patlak's slope.** The test-retest error is plotted as a function of the average [^18^F]FCho influx across the cell membranes. Black dots represent data points from the 3C2i; the black dash-dotted lines represent 95% confidence interval. The grey dots represent data points from the 2C1i; the grey lines represent 95% confidence interval. The black crosses represent data points from the Patlak plot; the black dotted lines represent 95% confidence interval.

To evaluate the robustness of the fitting of previously described models, the intra-observer variability was determined and depicted in a Bland-Altman plot (Figure 5). This plot corroborates that reduction of the number of compartments drastically reduces the percent error on the intra-observer variability. Consequently, the lowest intra-observer variability was obtained when graphical analysis, by means of the Patlak plot, was applied. It must be noted that the Patlak plot gave 0% error in several cases, while the other models (3C2i and 2C1i) almost never did.

**Figure 6:**
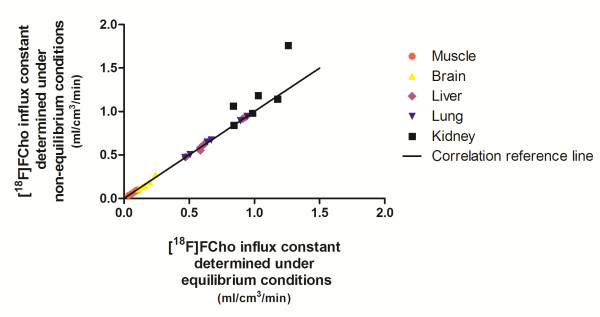
**A Spearman correlation plot depicting the [**^**18**^**F]FCho influx determined under equilibrium and non-equilibrium conditions.**

**Figure 7:**
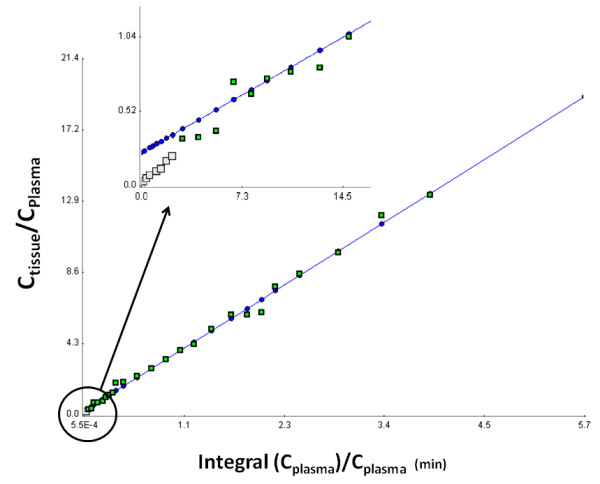
**A visualization of the Patlak model under equilibrium conditions.** The equilibrium conditions were obtained by selecting data points to obtain a linear regression. The grey and green squares represent the individual data points. The green squares are incorporated in the fitting under equilibrium conditions, whereas non-equilibrium fitting was performed on all the data points (i.e. grey and green data points). The fitting is represented by the blue line.

Since a robust fitting was obtained for the Patlak plot, it was investigated whether the tracer influx, determined by the slope of the linear regression, varied under equilibrium and non-equilibrium conditions. Several fittings indicated a good correlation between equilibrium and non-equilibrium conditions for all tissues, except the kidneys (Figure 6), thereby proving the insensitivity of the slope fitting in relation to the manual selection of the data points. Indeed, equilibrium conditions were selected by visual inspection and by including or excluding data time points to obtain a linear fit. Non-equilibrium conditions were obtained by including all data points in the linear regression (Figure 7).

## Discussion

Kinetic modelling requires arterial sampling which can be obtained by taking continuous or discrete blood samples. The latter is laborious, requires more animals due to the limited blood volume, and is characterized by complicated sampling intervals within a specific time window. Therefore, we chose to use the validated microvolumetric blood counter [9] to obtain continuous arterial blood samples by cannulation of the carotid artery by a precision operation and a syringe pump. The obtained blood curve was first smoothed in order to derive the first derivative, and then the dispersion constant was applied for dispersion correction, which occurred in the tubing during blood sampling.

Since only unbound tracer in plasma undergoes tissue uptake, a ratio of the plasma to blood concentration (*C*_pl_/*C*_bl_) was determined to convert the blood curve into a plasma curve. Therefore, standard hematocrit ranges [18] were used since the exact hematocrit and plasma volumes could not be determined after the addition of urea and sulphuric acid. Then, the discrete ratios were plotted and obtained a good fit using a one-exponential curve. It was preferred to use the equation obtained for blood-to-plasma corrections rather than a constant value, which would consequently underestimate plasma concentrations at early time points with perturbation of tracer uptake or unidirectional influx rate value (i.e. K_1_) as a result. In addition, the distribution of the plasma-to-blood concentration ratio indicates that [^18^F]FCho, as [^11^C]Cho [19], is accumulated into the red blood cells.

Comparing the blood metabolism of [^18^F]FCho to [^11^C]Cho [19], the following accordances were observed: (1) plasma extraction with chloroform proved that the amount of lipophilic [^18^F]FCho metabolites in the plasma (<5%, Table 1) is negligible, and (2) [^18^F]FBet was the only metabolite present in the plasma and it appeared almost immediately. Indeed, at 5 and 25 min post injection, 67.47% and 33.43% [^18^F]FCho could be detected, which correspond respectively to 62% and 18% [^11^C]Cho in the plasma [19] and to the metabolism pattern as described by Bansal et al. [10]. Therefore, we can conclude that fitting the percentage of the parent compound in the plasma to a one-exponential curve [20] provides a good fit (*R*^2^ = 0.9435). When using a three-exponential function as described by Roivainen et al. [19], fittings were not altered nor improved. Therefore, the one-exponential function was applied for metabolite corrections.

In general, kinetic models are developed as comprehensible models, which accurately describe the metabolic process or receptor binding, but are usually simplified to yield workable models that are able to quantify a parameter of interest. In oncology, this parameter is usually a specific enzyme or transporter, which can be described by a micro-parameter of the selected model. However, in a number of cases, the metabolic rate, which is a combination of micro-parameters, is determined, for example, glucose metabolic rate by [^18^F]FDG [21] or cell proliferation rate by [^18^F]FLT [15]. When evaluating the 3C2i model, the [^18^F]FCho influx across the cell membranes was calculated with corrections for (possible) [^18^F]FBet uptake, and the ratio of [^18^F]FCho to [^18^F]FBet uptake in tissues (*K*_1p_/*K*_1m_) was determined. The latter designates the magnitude of [^18^F]FBet uptake in the tissues and indirectly shows if additional corrections for [^18^F]Bet uptake should be implemented. These experiments showed that despite the explicit metabolic pattern, [^18^F]FBet uptake in the tissues is still limited, and so adjustments for its uptake should not be accounted for during kinetic modelling. Indeed, the [^18^F]FCho influx determined by the 3C2i model (which corrects for [^18^F]FBet uptake) and the sum of the [^18^F]FCho and [^18^F]FBet influx as determined by the 2C1i model were regarded as not statistically different (Figure 4). Furthermore, when comparing goodness-of-fit factors (i.e. AIC, SC, and MSC), no statistical enhancement (or superior fitting) could be detected by one of the models (i.e. 2C1i and 3C2i). It is worth noting that reducing the number of compartments, or in this case the input functions, consequently decreased the %COV of the micro-parameter estimates, resulting in reduced uncertainties. This was also observed in the intra-observer variability test. Therefore, we can conclude that the 2C1i is a workable kinetic model to estimate the [^18^F]FCho influx without creating additional errors.

Since [^18^F]FCho is a tracer which is assumed to be metabolically trapped in cells, including tumour cells [22], graphical analysis was applied to quantify the [^18^F]FCho influx across cell membranes. We evaluated if selection of the equilibrium time frame had an influence on the slope of the Patlak model, which is a measure of the [^18^F]FCho influx constant (i.e. K_i_). The Spearman correlation plot showed a good agreement for all tissues, except for kidney tissue. This might be due to the fact that the kidneys are responsible for tracer elimination, which affects the tracer accumulation and therefore might influence the fitting of the model. Since other tissues provided good correlations, we might conclude that non-equilibrium conditions do not influence the linear fitting for this radiotracer. These findings corroborate the Spearman correlation test which demonstrates the insensitivity of the [^18^F]FCho influx constant (i.e. K_i_) toward the selection of the linear portion. The fitting can thus be performed without selection of the equilibrium conditions which considerably simplifies the modelling process. Hence, graphical analysis, by means of the Patlak plot, is a very suitable way to estimate [^18^F]FCho influx across the cell membranes, except for kidney tissues. It is worth noting that the influx constant depends on imaging time, the type of tissue, and the type of tracer used, so preceding validation studies should be performed prior to implementation of the non-equilibrium data to the fitting process.

When comparing the [^18^F]FCho influx calculated by the 2C1i model (K_i_) with the Patlak slope, no statistical differences were observed (Kruskal-Wallis test), indicating a good agreement between both methods. On the other hand, the intra-observer variability test (Figure 5) favours the Patlak model, seeing that this analysis obtained the smallest percent error. The latter corresponds to the highest robust method for absolute quantification.

## Conclusions

Despite the early presence of the metabolite [^18^F]FBet in the blood, no explicit metabolite uptake was observed in the tissues. Consequently, modelling of the [^18^F]FBet uptake should not be performed during quantification of [^18^F]FCho uptake in tissues. [^18^F]FCho uptake is well described by the 2C1i and the Patlak model. Our preference goes to the latter since it is a reliable, precise, and robust method independent of scan time or plasma clearance. In addition, the Patlak plot could easily be implemented, even under non-equilibrium conditions and without creating additional errors, for the quantification of [^18^F]FCho uptake as, for example, in a therapy response assessment.

## Abbreviations

[18F]FCho: [^18^F]fluoromethylcholine; [18F]FBet: [^18^F]fluorobetaine; AIC: Akaike information criterion; MSC: Model selection criterion; SC: Schwartz criterion; SUV: Standardized uptake values.

## Competing interests

Both authors declare that they have no competing interests.

## Authors’ contributions

DS carried out the study concept and design, analyzed and interpreted the data, and wrote the manuscript. FDV coordinated and revised the manuscript. Both authors read and approved the final manuscript.
